# Acknowledgement to Reviewers of *Biomimetics* in 2018

**DOI:** 10.3390/biomimetics4010006

**Published:** 2019-01-17

**Authors:** Biomimetics Editorial Office

**Affiliations:** MDPI, St. Alban-Anlage 66, 4052 Basel, Switzerland

Rigorous peer-review is the corner-stone of high-quality academic publishing. The editorial team greatly appreciates the reviewers who contributed their knowledge and expertise to the journal’s editorial process over the past 12 months. In 2018, a total of 32 papers were published in the journal, with a median time to first decision of 25.5 days and a median time to publication of 48 days. The editors would like to express their sincere gratitude to the following reviewers for their cooperation and dedication in 2018:

Adil, Syed FarooqMartinez, Ramses V.Alam, Mohammad ParvezMartins, Luis R. M.Aljabali, AlaaMasouros, SpyrosAraromi, OluwaseunMendelson, LeahArdelean, Lavinia CosminaMohades Kasaei, Seyed MohammadrezaAydin, Yasemin O.Mohammed, Abdul SamadBall, VincentMorin, StephenBarth, Friedrich G.Mouchet, SébastienBax, N.A.M. (Noortje)Mutyala, KalyanBellardita, CarmeloMuzykantov, VladimirBelli, SemaNagel, Jacquelyn K.Benetatos, PanayotisNajarian, SiamakBerns, KarstenNishioka, YasutakaBhounsule, Pranav A.Oh, Dongyeop X.Bibb, RichardPapavassiliou, DimitriosBortot, ElianaPatil, MadhavBoschloo, GerritPereira, EduardoCarmona-Ribeiro, Ana M.Perinelli, Diego RomanoChander, HarishPezzella, AlessandroChen, LingxinPolygerinos, PanagiotisCho, Nam-JoonPoppinga, SimonCirera, JordiPrilutsky, BorisCrescenzi, OrlandoQuinn, RogerCulha, UtkuRai, PrakashDickerson, AndrewRamasamy, MohankandhasamyEdwards, Donald H.Rerat, MichelErgin, F. GökhanRodrigue, HugoFitl, PřemyslRokosz, KrzysztofFontanella, Chiara GiuliaRomero, EderFujiwara, TomokoSabaté, ManelGhosh, DebanjanaSadati, HadiGianneschi, NathanSamanta, DevleenaGiorgio-Serchi, FrancescoSchniepp, HannesGiraud, Marie NoëlleSchuster, BernhardGómez-Graña, SergioShafti, AliGudwin, Ricardo RibeiroSheng, JianHafuka, AkiraShepherd, Robert F.Hammond III, Frank LSherman, BenjaminHanafiah, Megat Ahmad Kamal MegatSigthorsson, David O.Hansma, HelenSong, Young MinHarnett, Cindy K.Steward, RobertHauser, HelmutSun, ZiqiHeepe, LarsTakaffoli, MahdiHoffmann, RolfTobias, GerardHong, SeonkiVallet-Regí, MaríaHumpolíček, PetrVan Der Zwaag, SybrandIjspeert, AukeVattikuti, Surya Veerendra PrabhakarIop, LauraVazquez Martinez, Juan ManuelIshikawa, TakujiVincent, Julian F. V.Jaudzems, KristapsVoigt, DagmarKaewpirom, SupraneeWalker, Ian D.Kang, RongjieWang, LiyuKhadiv, MajidWang, XiaoleiKlosterhalfen, BerndWei, QiKong, TiantianWeissburg, MarcKono, HitoshiWilliams, John A.Kramer-Bottiglio, RebeccaWilts, BodoKucinska-Lipka, JustynaWright, Cameron H. G.Kumagai, HiromuWysokowski, MarcinKunz, WernerXi, WeixianKyratsis, PanagiotisYoung, JohnLi, SuyiYudha, SalprimaLiebscher, JürgenZafar, MuhammadLiu, TingyiZelei, AmbrusLiu, YahuaZhang, HouxiangLondoño López, Martha ElenaZhang, TingnanLowe, AndrewZhang, YingyueLuo, ChengZhu, Quanmin

